# Treatment of Gonorrhœal Conjunctvitis

**Published:** 1881-12

**Authors:** 


### TREATMENT OF GONORRHŒAL CONJUNCTIVITIS.

One of the main indications consists in removing the pressure which the tense lids exert upon the eyeball, for the occurrence of corneal ulceration and gangrene depends largely upon the strangulation of vessels, thus produced. Some time ago Critchett relieved the tension and impending corneal danger in a very serious case by splitting the upper lid longitudinally and suturing the edges of the flaps thus formed to the skin of the brows. The procedure fulfills its object, but the risk seems imminent that shrinkage of the flaps may lead to subsequent deformity. The relief of the excessive tension has hence been attempted in another way by Fuchs (*Centralblatt fuer Augenheilkunde*, July, 1881). He splits the external commissure with the scissors, deepens the incision with a scalpel and prolongs it one centimetre beyond the external orbital rim dividing the soft tissues down to the bone. The upper lid can now be raised easily, the lower lid is kept everted by means of a loop suture until the swelling has subsided. During the operation hemorrhage occurs from the arteria zygomatico-orbitalis, which Fuchs thinks best to favor. The special advantage of the procedure apart from the relief of pressure is the thorough drainage of the pus. Two cases are given with incipient corneal change, the recovery of which proves the value of the procedure.—*Chicago Med. Review, October, 1881.*

